# Identification and clinicopathological analysis of potential *p73*-regulated biomarkers in colorectal cancer via integrative bioinformatics

**DOI:** 10.1038/s41598-024-60715-1

**Published:** 2024-04-30

**Authors:** Chanchal Bareja, Kountay Dwivedi, Apoorva Uboveja, Ankit Mathur, Naveen Kumar, Daman Saluja

**Affiliations:** 1 Dr. B.R. Ambedkar Center for Biomedical Research, University of Delhi, Delhi, 110007 India; 2 Department of Computer Science, Faculty of Mathematical Sciences, University of Delhi, Delhi, 110007 India; 3 Delhi School of Public Health, Institution of Eminence, University of Delhi, Delhi, 110007 India

**Keywords:** Transcriptomics, Integrative bioinformatics, P53, P73, TNM stage, Gene expression omnibus, Cancer, Computational biology and bioinformatics, Oncology

## Abstract

This study aims to decipher crucial biomarkers regulated by *p73* for the early detection of colorectal cancer (CRC) by employing a combination of integrative bioinformatics and expression profiling techniques. The transcriptome profile of HCT116 cell line *p53*^-/-^ *p73*^+/+^ and *p53*^-/-^ *p73* knockdown was performed to identify differentially expressed genes (DEGs). This was corroborated with three CRC tissue expression datasets available in Gene Expression Omnibus. Further analysis involved KEGG and Gene ontology to elucidate the functional roles of DEGs. The protein-protein interaction (PPI) network was constructed using Cytoscape to identify hub genes. Kaplan–Meier (KM) plots along with GEPIA and UALCAN database analysis provided the insights into the prognostic and diagnostic significance of these hub genes. Machine/deep learning algorithms were employed to perform TNM-stage classification. Transcriptome profiling revealed 1289 upregulated and 1897 downregulated genes. When intersected with employed CRC datasets, 284 DEGs were obtained. Comprehensive analysis using gene ontology and KEGG revealed enrichment of the DEGs in metabolic process, fatty acid biosynthesis, etc. The PPI network constructed using these 284 genes assisted in identifying 20 hub genes. Kaplan–Meier, GEPIA, and UALCAN analyses uncovered the clinicopathological relevance of these hub genes. Conclusively, the deep learning model achieved TNM-stage classification accuracy of 0.78 and 0.75 using 284 DEGs and 20 hub genes, respectively. The study represents a pioneer endeavor amalgamating transcriptomics, publicly available tissue datasets, and machine learning to unveil key CRC-associated genes. These genes are found relevant regarding the patients’ prognosis and diagnosis. The unveiled biomarkers exhibit robustness in TNM-stage prediction, thereby laying the foundation for future clinical applications and therapeutic interventions in CRC management.

## Introduction

Colorectal cancer (CRC) is the second leading cause of cancer-related deaths worldwide, claiming approximately 935,000 cancer deaths in 2020^1^. Its prevalence as the third most diagnosed cancer underscores a significant challenge to global public health systems, fueled by shortcomings in screening and treatment options^2^. Due to demographic shifts such as aging populations and sedentary lifestyles, an estimated 3.4 million new CRC cases are expected by 2040^3,4^. Urgent action is therefore required to bolster preventive measures and to advance treatment strategies to mitigate the impending rise in CRC cases and associated mortality.

The *p53* tumor suppressor gene is often subjected to frequent mutations in CRC and is aptly known as the “guardian of the genome”^5^. When activated in response to various stress signals^6^, including DNA damage or oncogene activation, the *p53* coordinates a multitude of downstream cellular responses, such as DNA repair, cell cycle arrest, senescence, metabolism, and cell death^7^. The *p53* functions primarily as a transcription factor controlling the expression of hundreds of target genes^8^. The *p73* transcription factor belongs to the *p53* family of tumor suppressors and bears substantial structural and functional similarity to the *p53*^9^. Like *p53*, *p73* is typically present at very low levels but is rapidly induced under genotoxic stress^10^. *p73* can bind to *p53* response elements and interact with *p53* target genes involved in cell cycle arrest and apoptotic cell death as well as activate the genes related to toxic stress response^10,11^. Furthermore, *p73* is known to activate target genes independently of *p53*^12^, and restoration of *p73* induces *p53*-like tumor suppressive effects^13^. Extensive investigation of *p73* status in primary human tumors shows that *p73* mutations are detected in less than 0.5% of human cancers, while more than 50% of cancers carry *p53* mutations^14^, making *p73* an attractive target for therapeutic intervention. However, a comprehensive dissection of the *p73* signaling axis needs to be elucidated to highlight its therapeutic efficacy (such as suppression of metastasis) in colorectal carcinoma^15^. Our prior investigations have substantiated the role of p73 as a transcription factor that exerts inhibitory effects on cancer cell invasion, migration, and metastasis. This inhibitory action is attributed to the direct binding of p73 to the Navigator-3 promoter, thereby modulating its expression levels^16^. Furthermore, our research has elucidated the involvement of p73 in the transcriptional regulation of the long non-coding RNA (lncRNA) FER1L4 in response to genotoxic stress^17^. In pursuit of a comprehensive understanding of the multifaceted targets of p73 during the carcinogenesis process, with the specific objective of identifying novel biomarkers for colorectal cancer (CRC) promotion, we employed integrative bioinformatics and machine learning. The landscape of biomedical research is undergoing a profound transformation, driven mainly by the advancement of technologies such as genomics and transcriptome sequencing, gene editing, and machine learning (ML). This transformative shift is progressively steering us from traditional medicine to precision medicine^18^. Among these technologies, next-generation sequencing (NGS) has revolutionized our ability to retrieve valuable information from DNA sequences, transcriptomics, and epigenetics by high-throughput sequencing at a fraction of time and cost as compared to conventional sequencing methodologies such as Sanger sequencing^19^. However, most NGS technologies are unable to precisely annotate the functions (specially those involving complex signaling pathways such as DNA repair and Wnt pathways) of differentially expressed genes (DEGs)^20^. Therefore, the combination of integrative bioinformatics methods and expression profiling technologies to overcome the hurdle of complex signaling pathways is pivotal^21^. Such an integrated approach can help in identification of appropriate biomarkers and pave the way for selecting systematic clinical strategies for prevention, diagnosis, and treatment options^22^. In this study, we conducted a comprehensive analysis of transcriptome profiles for HCT116 cells with distinct genetic characteristics: *p53*^-/-^ *p73*^+/+^ and *p53*^-/-^ *p73* knockdown (KD) cells to identify differentially expressed genes (DEGs). To substantiate these findings, we cross-checked our results with gene expression profiles of CRC patients obtained from the following NCBI Gene Expression Omnibus (GEO) datasets: GSE44076^23^, GSE110224^24^, and GSE113513^25^. Initially, we identified a set of key DEGs through the intersection of the genes present in the aforementioned GEO datasets and the transcriptome profile of the HCT 116 cell line. To gain a deeper understanding of these DEGs, we carried out Gene ontology (GO) and Kyoto Encyclopaedia of Genes and Genomes (KEGG) enrichment analysis^26–30^, which shed light on their biological functions and signal transduction pathways. Additionally, we created a protein-protein interaction (PPI) network using the STRING database^31^ and further extracted a set of hub genes with the help of the Cytohubba^32^ tool in CYTOSCAPE^33^. Moreover, the TCGA coloadenocarcinoma (COAD) dataset was utilized with the GEPIA^34^ and UALCAN^35^ online tools to analyze the expression profiles of the candidate (hub) genes, in addition to the pathological staging of the CRC patients. Further, the analysis of the prognostic behavior of the candidate genes was carried out using the KMPotter tool^36^. Finally, the TNM stage prediction of CRC patients via the identified DEGs and candidate hub genes was carried out to assess the predictive performance of the candidate genes. For this purpose, we implemented state-of-the-art machine learning and deep learning algorithms for the classification of TCGA-COAD samples into their accurate TNM stages. More specifically, we developed models based on extreme gradient boosting (XGBoost)^37^ and a deep neural network (DNN) for this classification. In essence, we combined bioinformatics and machine learning methods to analyze the pivotal genes associated with CRC. This research represents a pioneering combination of NGS, publicly accessible CRC tissue datasets, and machine learning algorithms to elucidate the role of *p53* and *p73* in colorectal cancer. Furthermore, based on the publicly available databases, we substantiated the diagnostic potential of these candidate genes. Collectively, our research findings deepens our understanding of the molecular mechanism underlying CRC, unravel novel molecular targets for diagnostic and therapeutic purposes, and provide improved long-term prognostic perspective for CRC patients.

## Materials and methods

### Cell line, culture conditions, and transfection

Cell line HCT116 *p53*^-/-^ was obtained from the lab of Bert Vogelstein, Johns Hopkins University, Maryland, U.S. The obtained cell line was cultured in Dulbecco Modified Eagle’s Medium (DMEM) containing 10% fetal bovine serum (Invitrogen) and 100 U/ml penicillin-streptomycin at 37 °C in humidified air with 5% CO_2_. HCT116 *p53*^-/-^ *p73* KD cell line was generated by transfecting a pBABEU6 vector containing shRNA targeting p73 (pooled puromycin-resistant population as previously described)^16^.

### Isolation, qualitative, and quantitative analysis of RNA, library preparation

RNA was isolated from samples by the Trizol method. The quality of the RNA was checked on a 1% formaldehyde denaturing agarose gel and quantified using a Nanodrop 8000 spectrophotometer. NGS library preparation and high-throughput sequencing were outsourced to Xcelris Labs Limited (Ahmedabad, Gujarat, India). The library was prepared using the Illumina TruSeq stranded mRNA library preparation kit. Briefly, mRNA was enriched from total RNA, followed by fragmentation. The fragmented mRNA was converted into first-strand cDNA, followed by second-strand generation, A-tailing, adapter ligation, and finally a limited number of PCR amplifications of the adaptor-ligated libraries.

### Quantity and quality check (QC) of the library on Bioanalyzer 2100 followed by cluster generation and sequencing

The amplified library was analyzed on a Bioanalyzer 2100 (Agilent Technologies) using a high-sensitivity DNA chip. After obtaining the Qubit concentration for the library and the mean peak size from the Bioanalyser profile, the library was loaded into the Illumina platform for cluster generation and sequencing. Paired-end sequencing allows the template fragments to be sequenced in both the forward and reverse directions. The library molecules bind to complementary adapter oligos on the paired-end flow cell. The adapters were designed to allow selective cleavage of the forward strands after re-synthesis of the reverse strand during sequencing. The copied reverse strand was then used to sequence from the opposite end of the fragment.

### Alignment, differential expression and Heatmap generation of differential genes in combination

Reference-guided transcript assembly was performed for all the samples, first by mapping HQ reads on the reference genome using HISAT2^38^ and then performing transcript assembly by StringTie^39^. A consensus set of transcripts was obtained using the StringTie merge function, which merges all the gene structures found in any of the samples. Transcript abundance was then estimated using merged transcript consensus again using StringTie and read counts thus obtained for each transcript were taken as input for differential expression analysis using the DESeq2^40^ package. A Python program was used to extract the read count information directly from the files generated by StringTie. Differential gene expression was inferred between sample groups by applying the R package. DESeq2, a bioconductor package is based on the negative binomial distribution method A list of transcripts was selected for heatmap generation based on the criteria that transcripts must be present in all four samples with the lowest *p*-value. The pheatmap package from R language was used for producing heatmaps. The color coding ranges from red to blue, where shades of red represent high transcript expression and shades of blue represent low transcript expression.

### Gene Ontology (GO) and KEGG pathway analysis

The Gene Ontology provides controlled vocabularies of defined terms representing gene product properties. These cover three domains: cellular component, the parts of a cell or its extracellular environment; molecular function: the elemental activities of a gene product at the molecular level, such as binding or catalysis; and biological process: operations or sets of molecular events with a defined beginning and end pertinent to the functioning of integrated living units: cells, tissues, organs, and organisms. For obtaining gene ontology for differentially expressed transcripts (DEGs) of transcriptome data, they were first annotated against the Uniprot, followed by mapping against UniprotKB^26^. GO and ortholog assignment and mapping of the differentially expressed transcripts to the biological pathways were performed using the KAAS^30^. Differentially expressed transcripts were compared against the KEGG database using BLASTX with a threshold bitscore value of 60 (default). Pathway analysis was performed using all differentially expressed transcript pathways using UniProtKB and KEGG-KAAS servers, respectively. WEB-based GEne SeT AnaLysis Toolkit (WebGestalt)^29^, is one of the most widely used gene set enrichment analysis tools that helps to extract biological insights from genes of interest. The over-representation analysis method was used for the KEGG and gene ontology analysis in terms of cellular components, biological processes, and molecular functions of intersected genes.

### NCBI-GEO for DEGs in CRC tissue and normal samples

NCBI-GEO (Gene Expression Omnibus) is a free database of microarray, gene, and NGS profiles. In this study, we tested GSE110224^24^, GSE113513^25^, and GSE44076^23^ to confirm the reliability of differentially expressed genes in transcriptome data. The microarray dataset contains suitable expression profiles of normal and CRC patients. GEO2R is the data file for the GEO processing tool. The difference is statistically significant and determined based on the classic t-test. considering 0<p-value<1 as the limiting criterion. In this study, we used GEO2R to filter the original data to identify DEGs and display them in Venny v2.1.

### STRING database and cytoscape tool to extract key hub genes

STRING^31^ aims to collect, store, and integrate all publicly available sources of protein-protein interaction (PPI) data and complement these with computational predictions of potential functions. We used STRING to develop and construct DEG-encoded proteins and PPI networks to analyze the interactions among candidate DEG-encoded proteins and visualize them with Cytoscape v3.7.2^33^. Finally, we utilized CytoHubba^32^, a plugin provided in Cytoscape, to extract a set of hub genes.

### UALCAN and GEPIA database for expression analysis

To analyze the expression profiles of DEGs as validation set in normal and tumor tissue samples along with different pathological stages, we utilized UALCAN^35^ and GEPIA^34^ databases. The former is a comprehensive web resource that provides analyses based on the The Cancer Genome Atlas Program (TCGA) and MET500 cohort data. In this study, we used UALCAN to analyze the expression profiles of DEGs in different stages of CRC and normal patients. The latter is the analysis tool containing RNA-seq expression data from 9736 tumors and 8587 normal tissue samples developed at Beijing University. In this study, we employed an expression analysis of genes in tumors and normal tissues by analyzing DEGs. The *p*-value cutoff was 0.05. A student’s t-test was used to generate *p*-values for expression analysis.

### KM PLOTTER for the prognostic value of key DEGs

KM PLOTTER^36^ is used to determine the association of key hub genes with the prognosis of CRC patients. We utilized the online KM plotter tool. The online repository provided a set of 1296 colon cancer patients and their associated overall survival profiles, where information for overall survival was available, using median expression levels for allotting patients into high and low groups. The survival period (in the number of days) and the probability of survival are indicated along the horizontal and vertical axes, respectively. The curve in orange color shows the instances with a high expression value of the gene for the specific (survival period in the number of days, survival probability) pair. Similarly, the curve in black color shows the instances with a low expression value of the gene for the specific (survival period in the number of days, survival probability) pair.

### TNM stage prediction performance of the identified DEGs and Hub genes

#### Dataset details

To evaluate the TNM stage prediction performance of the identified sets of DEGs, i.e., the set of 284 common DEGs and the set of 20 Hub genes, the *coloadenocarcinoma* dataset generated by the TCGA Research Network was utilized. The TCGA-COAD, comprised the pan- cancer-normalized RNA-Seq gene expression transcriptomics data of 577 colorectal adenocarcinoma samples; each sample was described by the values of a set of 20,531 genes. The RNA-Seq gene expression value of these genes was computed using the IlluminaHiSeq platform and was subsequently mean-normalized (per gene) across all the TCGA cohorts. The dataset was downloaded from cBioportal in September 2023.

#### Dataset preprocessing

The samples in the TCGA-COAD dataset were categorized into granular TNM stages as shown in Table 1. The dataset was found relatively imbalanced in the samples in each granular TNM stage. The granular stages corresponding to distinct TNM stages were combined to overcome this issue, resulting in the following final stages- Stages I, II, III, and IV. Subsequently, the Synthetic Minority Oversampling Technique-Tomek (SMOTETomek)^41,42^ augmentation technique was leveraged to finally balance the resultant dataset after combining the substages into their respective stage. Being a data augmentation technique, the SMOTETomek utilizes SMOTE for oversampling and Tomek links for under-sampling. The SMOTE is an augmentation technique that selects instances closer to each other in the feature space, draws a line between the instances in the feature space, and selects a new instance at a point along that drawn line. However, it often generates noisy instances by interpolating new instances between marginal outliers and inliers. To resolve this issue, Tomek’s link cleans the space generated by SMOTE during over-sampling. The final dataset considered for prediction analysis comprises the set of instances per stage as shown in the fourth row (SMOTETomek-augmented Combined-Stage Instances) of Table 1.

**Table 1:** Stage-wise classification of the instances in TCGA-COAD.

TNM stages	I	II	IIA	IIB	IIC	III	IIIA	IIIB	IIIC	IV	IVA	IVB
Inital instances	102	35	171	12	2	22	14	80	54	57	26	2
Combined-stage instances	STAGE I (102)	STAGE II (220)				STAGE III (170)				STAGE IV (85)		
SMOTETomek-augmented combined-stage instances	STAGE I (210)	STAGE II (212)				STAGE III (218)				STAGE IV (218)		

#### ML/DL models architecture

Two state-of-the-art models were developed to perform a comparative analysis of the TNM stage prediction of the COAD samples using the identified DEGs-the first one being the XGBoost model and the second one being a deep neural network. The max depth of the XGboost model was kept to 3, the learning rate was kept to 0.1, and the maximum number of estimators was kept at 100. Since the TNM stage classification is a multi-class classification problem, the objective function of the XGBoost model was kept as “softmax” with a number of classes set as four. On the other hand, the developed deep neural network comprised an input layer of size equal to the number of genes in the identified DEGs, i.e., 284 for the common DEGs and 20 for the hub genes. Next, the network was composed of a set of four hidden layers, each of size 1024. Finally, the output layer of the network comprised four nodes, followed by a softmax layer for multi-class classification. Each hidden layer was followed by a dropout layer with successive “keep-probability” of 0.3, 0.2, 0.1, and 0.1, respectively. The activation functions used were ReLU and LeakyReLU.

## Results

### Transcriptome Analysis of HCT116 cell line: mapping and alignment

The high-quality reads of duplicate Controls (Control_rep1 and Control_rep2) and *p73* KD (KD_rep1 and KD_rep2) samples were aligned to the Homo sapiens genome utilizing the HISAT2 tool. This process facilitated the extraction of read subsets associated with each gene, which were subsequently assembled and used for transcript quantification. Notably, approximately 93% of reads were successfully mapped to the reference genome in each sample. The mapping statistics are shown in Table 2.

**Table 2:** Reads mapping statistics.

Sample	Total reads (R1+R2))	No. of mapped reads	% of mapped reads
Control_rep1	147,631,742	143,445,281	97.16
Control_rep2	83,841,690	80,626,187	96.16
KD_rep1	102,840,390	100,094.019	97.33
KD_rep2	64,133,350	60,157,642	93.80

The StringTie assembly tool resulted in 121,178 and 94,028 transcripts in Control_rep1 and KD_rep1 samples, respectively. Similarly, for Control_rep2 and KD_rep2, it resulted in 81,255 and 71,980 transcripts, respectively. To merge the obtained transcripts from each of the aforementioned samples, the merge function of the StringTie tool was used, which resulted in 200,815 transcripts (Merged GTF). The statistics of merged transcripts and individual transcript assembly are shown in Table 3.

**Table 3:** Statistics of transcript assembly.

Sample name	#Assembled transcripts
Merged GTF	200,815
Control_rep1	121,178
Control_rep2	81,255
KD_rep1	94,028
KD_rep2	71,980

### Differential transcript analysis, heat map and volcano plot of DEGs

**Figure 1:**
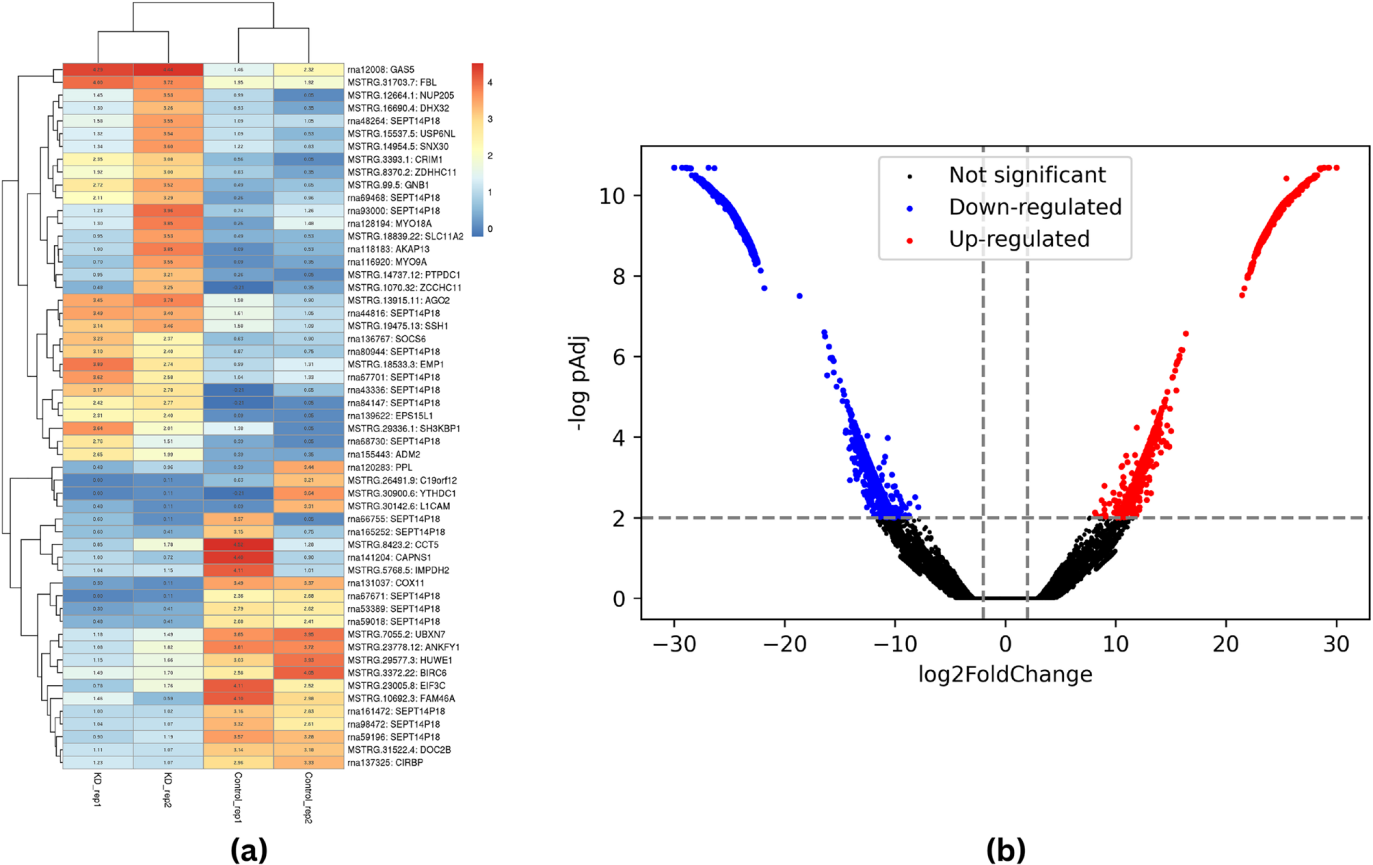
Differentially expressed transcript profile of HCT116 cell line. (**a**) shows the heatmap representing the most significant genes expressed in all four samples plotted using log_10_ of normalized read count values for HCT116*p53*^-/-^ *p73*^+/+^ and *p53*^-/-^ *p73* knockdown (KD) cell line, where shades of blue represent downregulated genes and shades of red represent highly expressed genes. Further, (**b**) depicts the volcano plot of the distribution of expressed transcripts. The red and blue color correspond to significantly up and downregulated transcripts respectively with adjusted *p*-value < 0.05 and the black color corresponds to non-significant transcripts with adjusted *p*-value > 0.05.

The prepDE.py tool was utilized to extract the read count information from the files generated by the StringTie tool. For the analysis of differential expression, a group-wise comparison was made between the Control and the *p73*KD groups. The DEGs were inferred between sample groups via the DESeq2 (v1.26.0) package. A total of 137,713 genes are differentially expressed in all of the two combinations. Among these genes, 1,289 were upregulated (log_2_ FC > 0, *p* < 0.05) while 1897 were downregulated genes (log_2_FC < 0, *p* < 0.05) exhibiting significant differential expression. Using the pheatmap package in R software, we generated a heatmap illustrating the 50 most significant DEGs, encompassing highly upregulated and downregulated genes (Fig. 1a). The heatmap was constructed based on the log_10_ -transformed values of the normalized read counts for both the control and the *p73* KD samples. In the heatmap, the shades of blue represent the downregulated genes, while the shades of red represent the upregulated genes. In addition, Fig. 1b represents the volcano plot of the DEGs arranged along dimensions of biological as well as statistical significance. The red and blue color in the volcano plot corresponds to upregulated and downregulated transcripts respectively with adjusted *p*-value < 0.05, and the black color corresponds to non-significant transcripts with adjusted *p*-value > 0.05.

### Functional analysis of the differential transcripts

**Figure 2:**
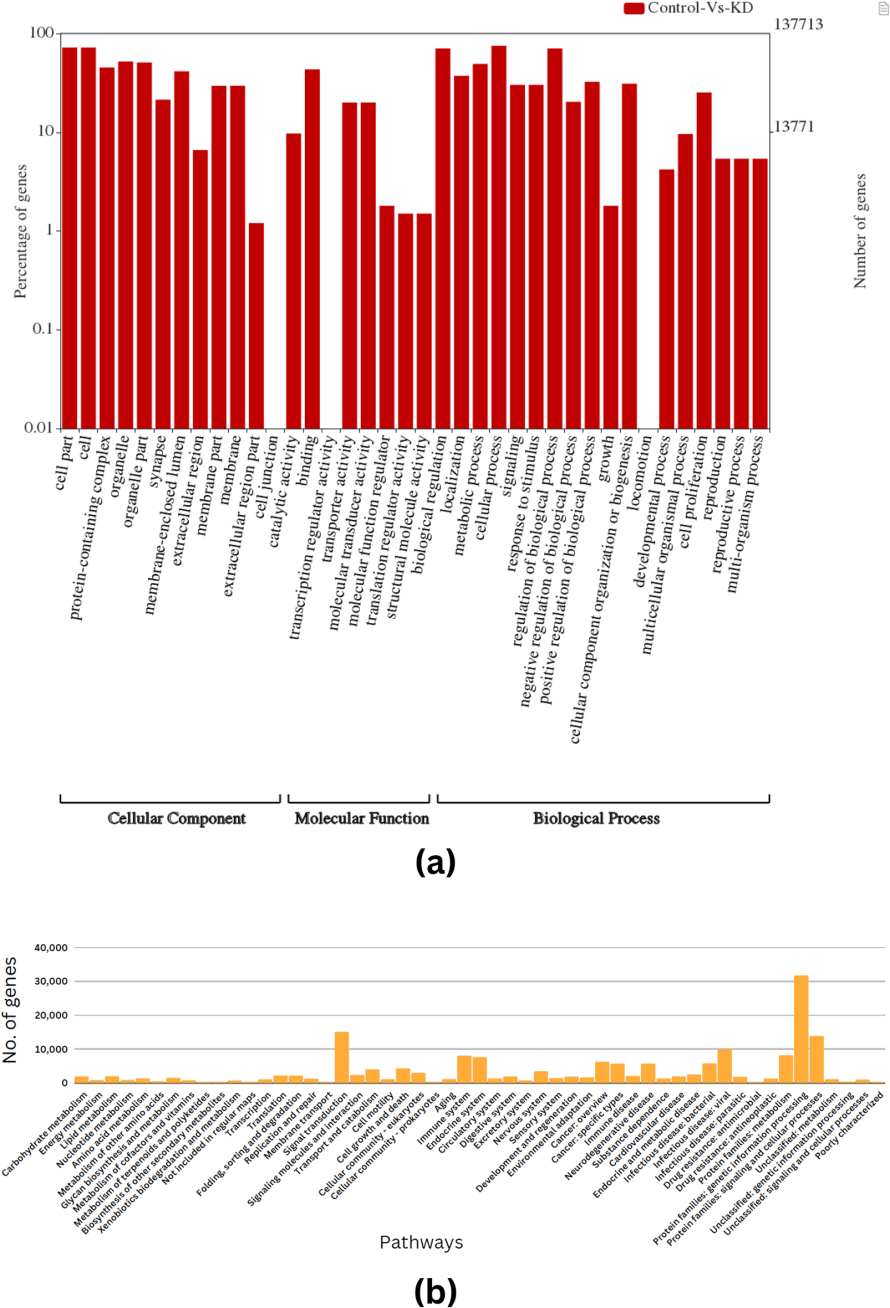
Functional clustering of the differentially expressed transcriptome profile of HCT116 cell line in control and knockdown samples as per GO terms. Figure 2a represents GO distribution for differentially expressed transcripts encompassing Biological Process (BP); Molecular Function (MF); Cellular Component (CC) along the x-axis and percentage and number of genes along the y-axis. Figure 2b shows biological pathways for differentially expressed transcripts via KAAS^27,28^.

To obtain the gene ontology for the differentially expressed transcripts, the Uniprot database is utilized, followed by mapping against UniprotKB. A set of 96,513, 104,594, and 92,249 genes were found to be significantly enriched in seventeen biological processes, thirteen cellular components, and eight molecular functions, respectively, as depicted in Fig. 2a. For the ortholog assignment and mapping of the differentially expressed transcripts to the biological pathways, the Kyoto Encyclopedia of Genes and Genomes (KEGG) Automatic Annotation Server (KAAS) was utilized. The differentially expressed transcripts were compared against the KEGG database using the BLASTX program with a default threshold bit-score value of 60. A set of 9191 transcripts was found to be significantly enriched in the metabolic pathways of major biomolecules such as carbohydrates, lipids, nucleotides, amino acids, glycans, cofactors, vitamins, terpenoids, and polyketides. Further, these transcripts were found to be involved in metabolism, genetic information processing, environmental information processing, and cellular processes. A total of 14,926 and 6,109 DEGs were found to be contributing to the activities of the signal transduction and cancer pathways, respectively. Figure 2b shows the number of genes mapped to the particular pathways.

### Identification of common DEGs among transcriptome and gene expression omnibus datasets

**Figure 3:**
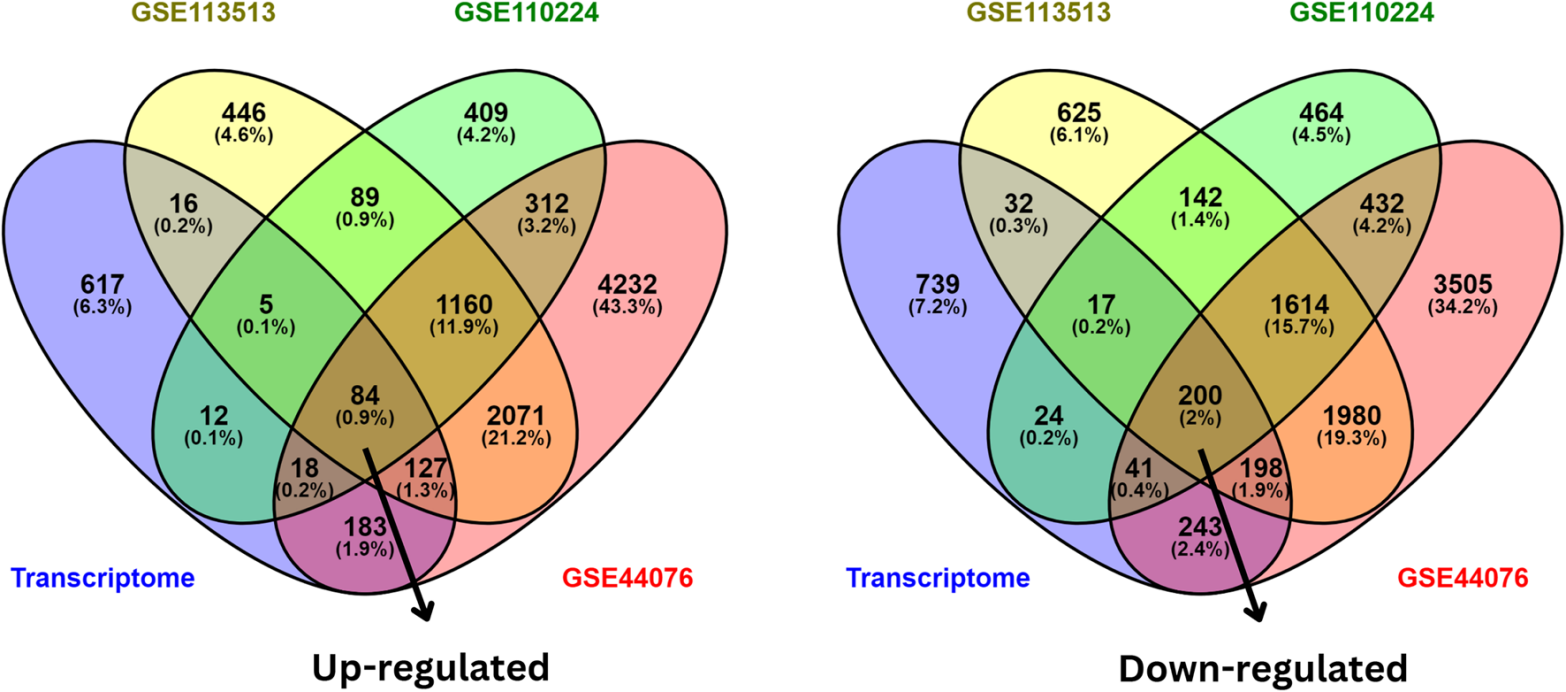
Venn diagram was visualized in Venny v2.1 tool, showing a total of 284 intersected genes among GEO datasets and Transcriptome data of HCT116 cell line. (**a**) represents the intersection of upregulated genes and (**b**) represents downregulated genes obtained after the cross-checking of transcriptome data with mentioned GEO datasets.

This study employs three Gene Expression Omnibus (GEO) datasets, namely GSE44076, GSE110224, and GSE113513, and the transcriptome data for cell lines HCT116 *p53*^-/-^ *p73*^+/+^ and HCT116 *p53*^-/-^ *p73* KD. We screened the microarray data of primary CRC tissue samples from the aforementioned GEO datasets as a preprocessing step. The GSE110224 comprised the expression profiling of 34 samples based on the GPL570 platform, including 17 adjacent normal and 17 primary colorectal adenocarcinoma samples. The GSE44076 comprised colon tumor samples from 98 patients and adjacent paired normal mucosa samples from 50 healthy donors obtained using the platform GPL13667 (Affymetrix Human Genome U219 Arrays). The GSE113513 dataset contained 14 colorectal cancer tissues and 14 normal tissue samples. We used the GEO2R method for preprocessing and considered a set of genes with *p*-value < 0.05 as significantly differentially expressed genes from the GEO datasets. Conclusively, we leveraged the Venny v2.1 tool to identify 284 consistent genes that are found in the intersection of all three GEO datasets and the transcriptome data of the HCT116 cell line. These 284 genes included 84 upregulated and 200 downregulated genes as shown in Fig. 3.

### Enrichment analysis of common DEGs among transcriptome data and GEO datasets

**Figure 4:**
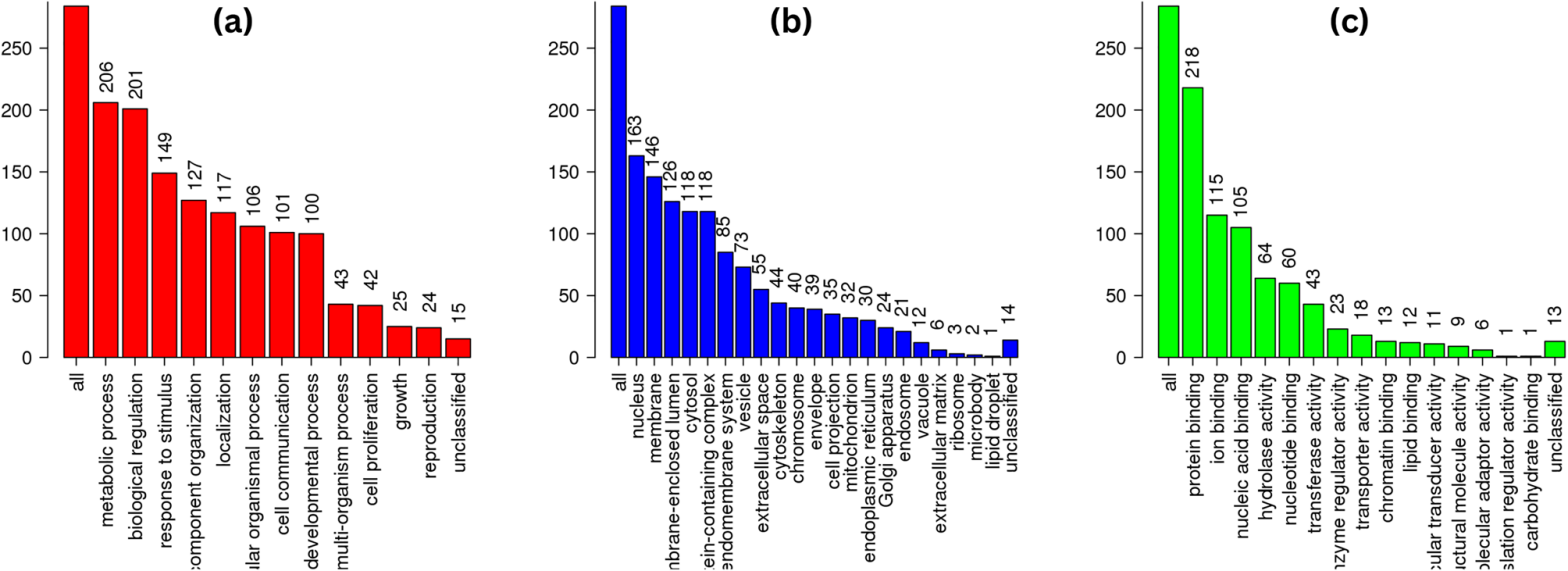
The enrichment analysis of 284 DEGs in CRC. (**a**) Bar chart of GO enrichment in biological process terms; (**b**) cellular component terms; and (**c**) molecular function terms.

**Figure 5:**
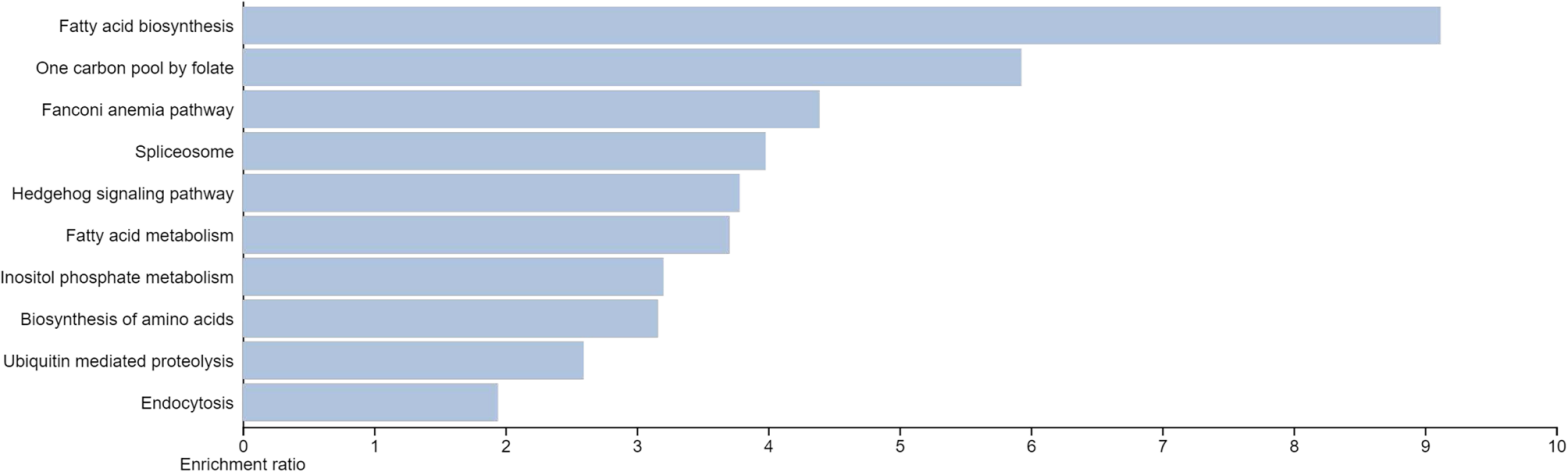
KEGG pathway^27,28^ enrichment in 284 intersected DEGs.

The obtained DEGs were analyzed for functional enrichment (GO and KEGG pathways) by using the WEB-based GEne SeT AnaLysis Toolkit (WebGestalt). The DEGs were found to significantly enrich in various biological processes such as metabolic processes, biological regulation, response to stimulus, cellular component organization, cell communication, developmental processes, multi-organism processes, cell proliferation, growth, and reproduction represented in Fig. 4a. Subsequently, the cellular components found to be enriched were the nucleus, membrane, membrane-enclosed lumen, cytosol, protein-containing complex, endomembrane system, vesicle, extracellular space, cytoskeleton, chromosome, envelope, cell projection, mitochondrion, endoplasmic reticulum, and Golgi apparatus (Fig. 4b). Moreover, the molecular functions found enriched were protein binding, ion binding, nucleic acid binding, hydrolase activity, nucleotide binding, transferase activity, enzyme regulator activity, chromatin binding, lipid binding, molecular transducer activity, structural molecular activity, molecular adaptor activity, translation regulator activity, and carbohydrate-binding (Fig. 4c). Figure 5 depicts the analysis of the biological pathways showing that 284 DEGs are mainly enriched in Fatty acid biosynthesis, one carbon pool by folate, Fanconi anemia pathway, spliceosome, hedgehog signaling pathway, fatty acid metabolism, inositol phosphate metabolism, biosynthesis of amino acids, ubiquitin-mediated proteolysis, and endocytosis.

### PPI network for central hub genes identification

**Figure 6:**
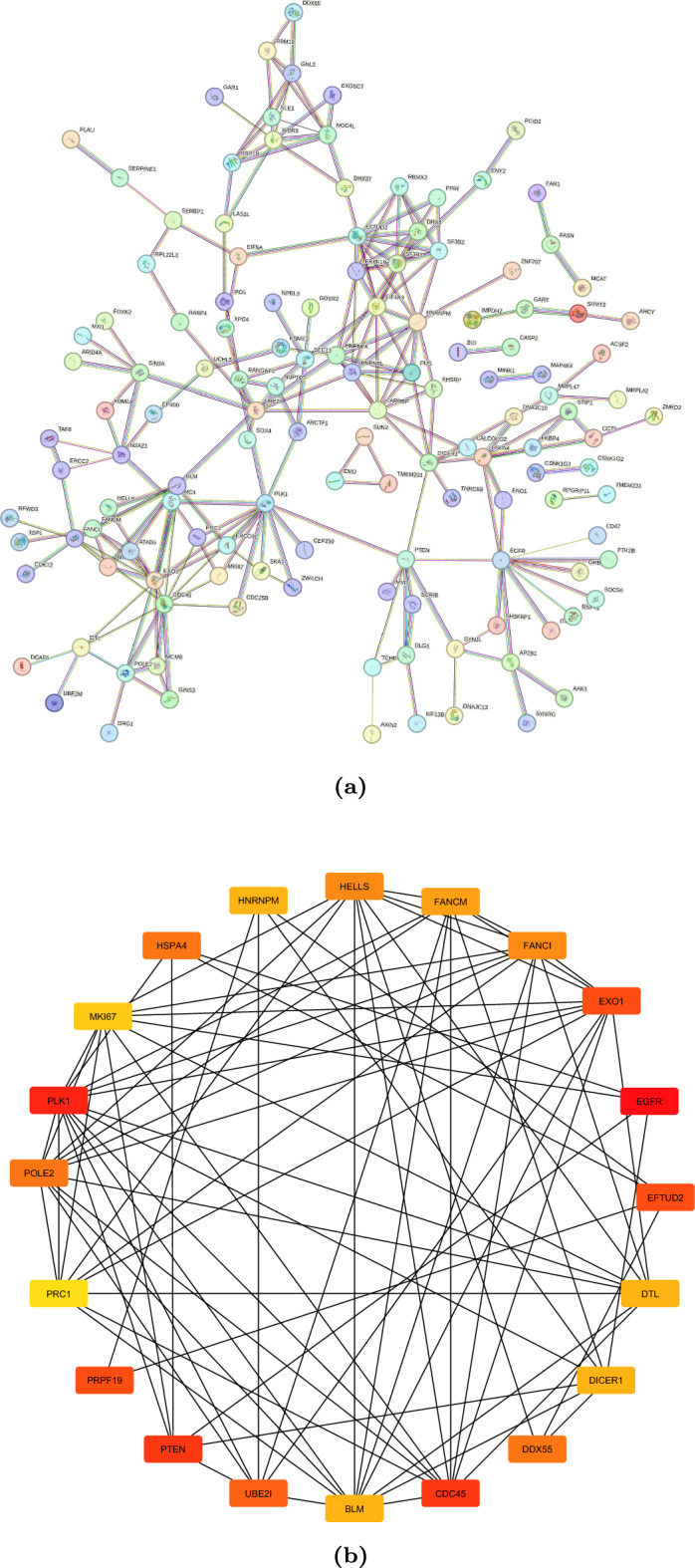
PPI network composed of 284 DEGs (**a**). For the 20 hub genes calculated by Cytoscape software; the red represents the degree of connectivity. The deeper the red, the higher the degree of connectivity shown in (**b**).

Figure 6a shows the protein-protein-interaction (PPI) network constructed for the 284 intersected genes using the STRING database and Cytoscape v3.6.0 software. A high-confidence interaction score $>0.7$ was considered to build the network. With the help of this PPI network, a set of twenty central hub genes with maximum connectivity with the rest of the nodes is identified and visualized in the CytoHubba tool in Cytoscape as presented in Fig. 6b. *PRC1, HNRNPM, DTL, FANCI, EXO1, UBE2I, DICER1, PTEN, PRPF19, CDC45, MKI67, EFTUD2, BLM, POLE2, FANCM, DDX55, HELLS, HSPA4, PLK1*, and *EGFR* are twenty key hub genes extracted from PPI network of 284 intersected genes via cytoscape.

### Gene expression analysis of the central hub genes

**Figure 7:**
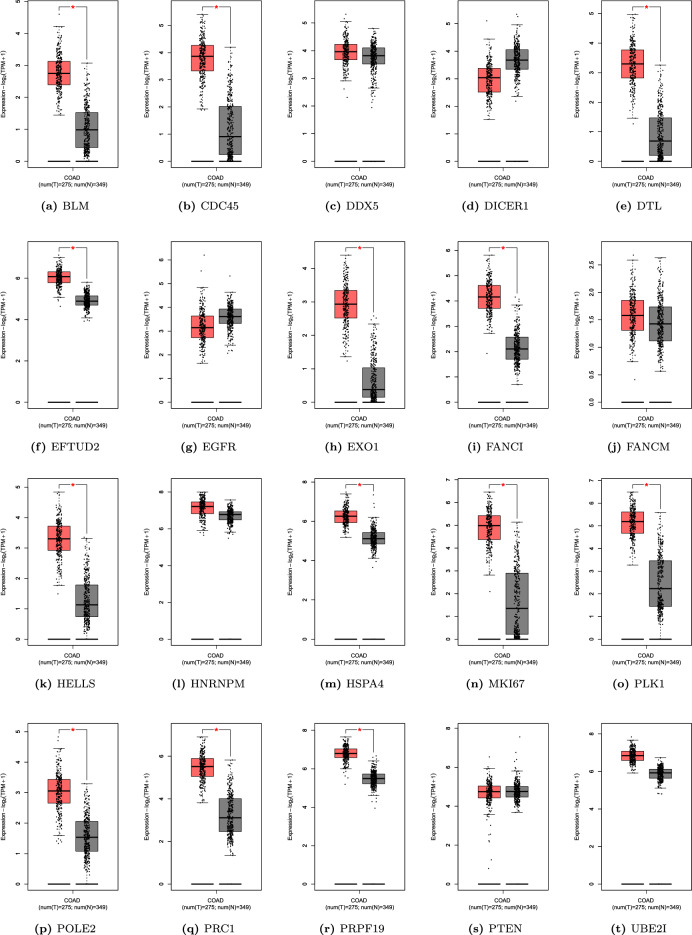
(**a**–**t**) Gene expression analysis of 20 key hub genes in COAD patients from TCGA in comparison to normal patients based on GEPIA database.

**Figure 8:**
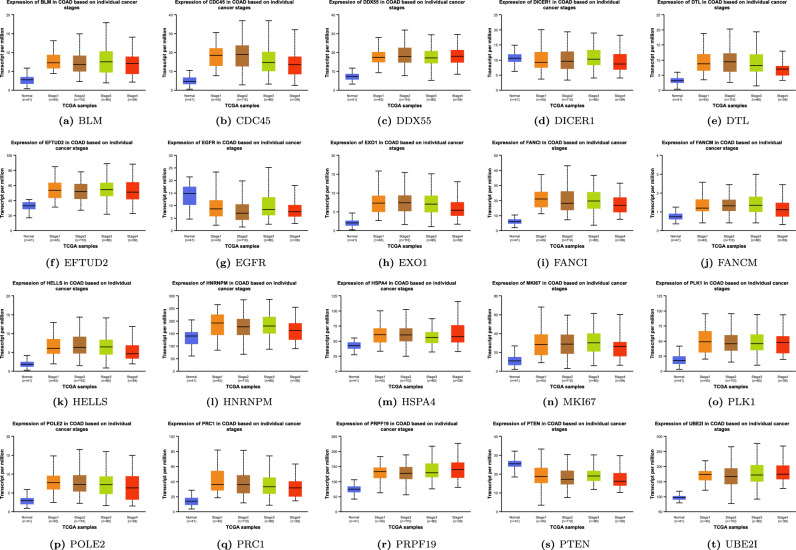
(**a**–**t**) Expression profile of 20 key hub genes in normal patients and colon cancer, stratified based on stage criteria analyzed via UALCAN.

We used the GEPIA database to analyze the expression of twenty candidate genes in cancer tissues and normal samples from the TCGA COAD dataset. The results show that *BLM, CDC45, DTL, EFTUD2, EXO1, FANCI, HELLS, HSPA4, MKI67, PRPF19, PLK1, POLE2, PRC1* were all significantly upregulated in tumors in comparison to normal tissues presented in Fig. 7a–t. Additionally, the UALCAN database was used to analyze the expression of all key hub genes in the pathological staging of COAD. We found that the expression of all the discovered candidate genes varied significantly between different stages and the adjacent normal tissue, except *DICER1* shown in Fig. 8a–t. Additionally, we observed that the gene expression of *EGFR* varied significantly between Stages I, II, and IV, and the normal samples (*p*<0.05).

### Overall survival analysis

**Figure 9:**
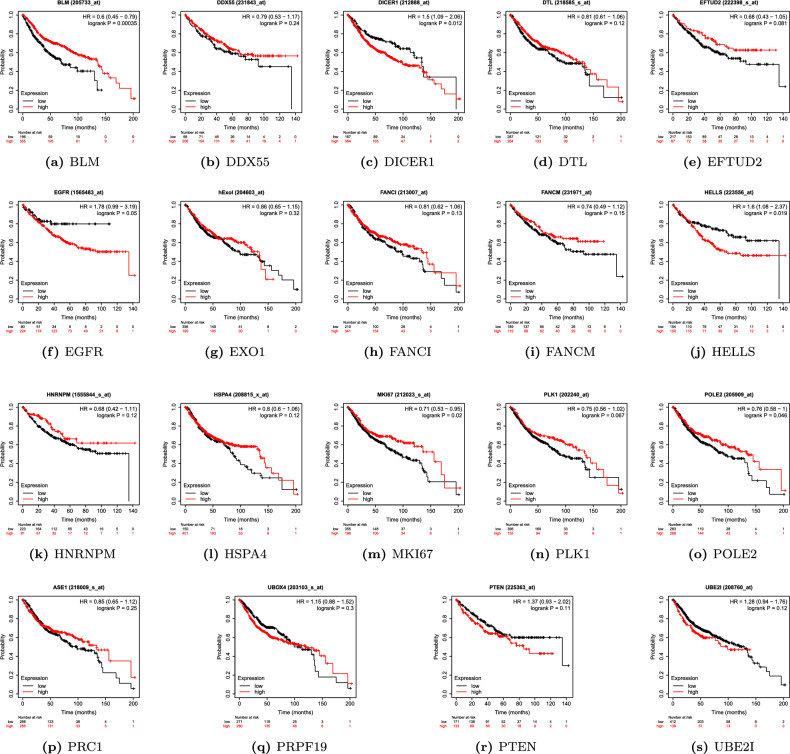
Kaplan–Meier curves of key 19 genes obtained via KM Plotter. The survival period (in the number of days) and the probability of survival are indicated along the horizontal and vertical axes, respectively.

To determine the association of key hub genes with the prognosis of CRC patients, we utilized the online KM plotter tool^36^. The online repository provided a set of 1296 colon cancer patients and their associated overall survival profiles. where information for overall survival was available. We performed survival analysis by constructing a Kaplan–Meier plot for all 20 hub genes obtained from Cytoscape, using median expression levels for allotting patients into high and low groups. The survival period (in the number of days) and the probability of survival are indicated along the horizontal and vertical axes, respectively. The curve in orange shows the instances with a high expression value of the gene for the specific (survival period in the number of days, survival probability) pair. Similarly, the curve in black color shows the instances with a low expression value of the gene for the specific (survival period in the number of days, survival probability) pair. Based on Kaplan–Meier curves with log-rank p-value, *BLM, DICER1, HELLS, EGFR, MKI67*, and *POLE2* were all associated with the overall survival of patients (Fig. 9). Thus, these genes established their importance in prognostic evaluation by segregating the high survival probability group from the low survival probability group, based on the differences in the expression level.

### TNM stage classification results of TCGA-COAD

**Figure 10:**
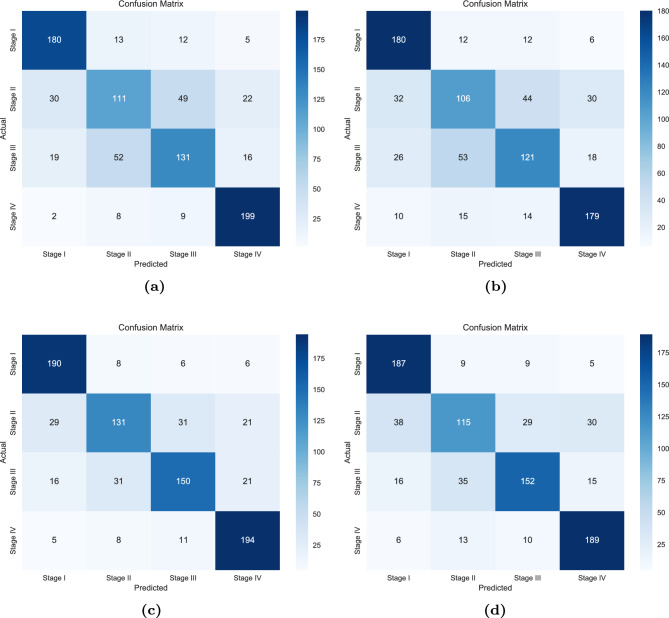
Confusion matrices of the respective *XGBoost* and *Neural Network* models. The order of the confusion matrices are as follows: Fig. 10a XGBoost trained on the 284 intersected genes, Fig. 10b *XGBoost* trained on the 20 hub genes, Fig. 10c *Deep Neural Network* trained on the 284 intersected genes, and Fig. 10d *Deep Neural Network* trained on the 20 hub genes. It should be noted that although the proposed neural network achieves better accuracy when the 284 intersected genes are passed as the feature set, the variance of the neural network-based model trained on the 20 hub genes is relatively less than the other trained models.

**Figure 11:**
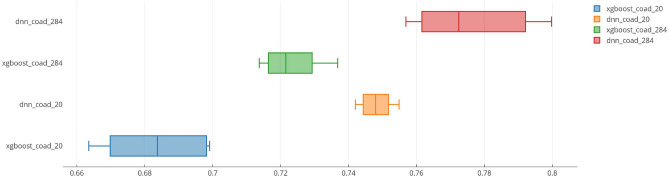
Boxplot of the classification performance of *xgboost*-based model and the *dnn*-based model on different datasets under study.

The classification performance of the developed machine learning model, i.e., XGBoost, and the deep learning model, i.e., deep neural network was evaluated on the basis 10-fold cross-validation method at a 95% confidence interval. Table 4 shows the classification performance of both models. For convenience, the TCGA-COAD dataset with only the hub genes as a feature set was named “*COAD_20*”, and the TCGA-COAD dataset with only the 284 common DEGs as a feature set was named “*COAD_284*”. Moreover, the XGBoost-based model and the deep neural network-based model were appropriately named as “*xgboost*” and “*dnn*”, respectively. Further, the confusion matrix of the models for both datasets is shown in Fig. 10, while the box plot is shown in Fig. 11. It can be observed that the classification performance of the *dnn* model when trained on the *COAD_284* dataset yields the best accuracy (0.78 ± 0.009). Nevertheless, by observing the boxplot, it is concluded that the variance in the classification accuracy (0.75 ± 0.002) is the least when the *dnn* model is trained on the *COAD_20* dataset.

**Table 4:** Comparison of the stage-wise classification performance of the *xgboost*-based and the *dnn*-based model. It is observed that the *dnn*-based model performs relatively better than the *xgboost*-based model in both *COAD_20* and *COAD_284* dataset.

Dataset	10-fold cross-validation Accuracy (95% C.I.)	
	*xgboost*	*dnn*
*COAD_20*	0.68 ± 0.008	0.75 ± 0.002
*COAD_284*	0.72 ± 0.004	0.78 ± 0.009

## Discussion

According to the SEER 2022 database, a staggering 36% of CRCs are diagnosed after metastasis at distal sites, resulting in poor prognosis and therapy response^43^. While, mutations in the *p53* gene have long been associated with the early onset of CRCs, a more diversified role of its family gene *p73 *has emerged recently^44^. The pleiotropic function of *p73 *in carcinogenesis emphasizes its potential as a key gene for in-depth study and targeting to address multiple facets of tumor development^45^.

In the current study, we used an in vitro cellular model system with sequential deletions of *p53* and *p73* genes. Through differential gene expression analysis comparing *p53*^-/-^ and p73kd cell lines by NGS, we identified crucial regulatory genes associated with diverse biological processes. To validate these findings, we cross-referenced them with three independent CRC GEO datasets, enabling a deeper comprehension of the *p73* gene regulatory network. We identified 284 common DEGs among transcriptome data and the three GEO datasets, including 84 upregulated and 200 downregulated genes. These DEGs were predominantly involved in metabolic processes and other biological regulations. Consistent with the established understanding that mutations and/or deletion in tumor suppressors affect cellular stress responses, including metabolic reprogramming^45,46^, we observed a pronounced influence on the fatty acid biosynthesis pathway and folate co-factor-mediated pathways. These pathways are known to play pivotal roles in multiple physiological processes, including purine biosynthesis^47^, amino acid homeostasis^48^, redox defense^49^, and epigenetic maintenance^50^. These observations corroborated the transcriptome analysis, where protein families associated with cellular metabolism and signaling pathways were highly affected by *p53*/*p73* deletions, indicating the crucial role of *p73* in regulating cellular metabolism. Furthermore, genes associated with Fanconi anemia, a condition characterized by inherited bone marrow failure, were predominantly affected. Fanconi Anaemia is majorly regulated by more than 23 FA complementation genes (FANC) involved in DNA repair pathways^51^. Evidence suggests that mismatched repair genes (MMR) involved in homologous recombination (HR) repair play an essential role in CRCs^52,53^. Additionally, a direct interaction of MMR proteins and some FA proteins has also been identified^54^ suggesting a strong correlation between the FANC gene and increased risk of CRC. To gain an in-depth analysis of the central genes among the 284 identified DEGs, we extracted a set of twenty hub genes. Notably, FANC was found to be among the 20 identified hub genes, suggesting a strong correlation between *p73 *and FA genes. It is interesting to note that 11 out of 20 hub genes *(HELLS, FANCM, FANCI, EXO1, EFTUD2, DDX55, BLM, PRPF19, POLE2, MKI67, HNRNPM)* were explicitly associated with DNA replication and repair pathways, while five genes *(EGFR, DTL, CDC45, PRC1, PLK1)* were involved in cell proliferation. The dysregulated ATM-chk2-p53 axis is known to be involved in aberrant DNA repair machinery, and may promote genomic instability^55^. Our study suggests that *p73* could potentially influence crucial DNA repair pathways to compensate for the vital role of *p53* in tumors with *p53* deletions. Moreover, in line with the well-documented effects of *p53* mutations on several aspects of cell proliferation such as cell cycle arrest, mitotic spindle stabilization, and suppressing spindle assembly checkpoints^56^, we found similar alterations in hub genes (*EGFR, DTL, CDC45, PRC1, PLK1*) that are associated with cell proliferation upon *p73* knockdown. Our results also indicate high to moderate impact, with *p*<0.05 (*BLM, DICER1, EFTUD2, EGFR, HELLS, MKI67, PLK1, POLE2*) to moderate impact, with *p*>0.05 (*DDX55, DTL, EXO1, FANCI, FANCM, HNRNPM, HSPA4, PRC1, PTEN, UBE2I, UBOX4*) on survival outcomes of these 20 hub genes in CRC patients. Nearly all the 20 hub genes were found to be differentially altered at every stage in Coloadenocarcinoma patients, strongly suggesting an analogous role for *p53* and *p73* in regulating diverse functions at nearly every stage of carcinogenesis. These findings underscore the significance of both *p53* and *p73* in the multifaceted process of cancer development and progression. It is crucial to emphasize that while the GEO datasets utilized in the study lack the capability to definitively ascertain the precise downregulation of p53/p73 genes at an individual patient level, the overarching analysis of total gene expression unequivocally validates a significant downregulation of these genes. To corroborate the findings of our study, it is imperative to undertake additional *in vitro* analyses, especially delving into the regulatory mechanisms of the identified hub genes under diverse p53/p73 statuses.

Prior research has predominantly focused on CRC classification (molecular or stage-wise) using imagery data such as histopathological images^57–59^. However, given that cancer fundamentally manifests as a genomic disease, its intrinsic molecular attributes can be precisely captured using omics-based data, notably transcriptomics data. To the best of our knowledge, this study represents the pioneering efforts in utilizing RNA-Seq gene expression transcriptomics data to perform a TNM stage prediction analysis utilizing a set of 284 common DEGs and the 20 hub genes. Leveraging the state-of-the-art XGBoost and deep neural network models on the TCGA-COAD dataset, the deep neural network model significantly outperformed the competitive XGBoost model when both the set of 284 common DEGs and the 20 genes were used as the input feature sets. The analysis concludes with the observation that the identified DEGs and the hub genes are significantly efficacious when utilized by an AI agent for TNM stage prediction in a CRC patient.

## Conclusion

Our study provides intricate transcript profile of colorectal cancer cell lines with distinct genetic strains uncovering notable differences. Furthermore, through the integration of diverse datasets of CRC patients, we identified key hub genes capable of accurately classifying the CRC patients into their appropriate TNM stages. Notably, our research highlights the efficacy of *p73* in regulating the expression of a plethora of genes marking a milestone in the biomarker discovery for the early and effective diagnosis and prognosis of CRC patients. The current findings hold promise for the development of significant therapeutic interventions for CRC patients (Supplementary Files S1, S2).

### Supplementary Information

Supplementary Information 1.

Supplementary Information 2.

## Data Availability

The data sets used and/or analyzed during the current study are available from the corresponding author upon reasonable request. The data have been made available on GitHub for the convenience of our readers. The relevant codes for the transcriptome analysis and machine learning algorithms are provided in the following GitHub repository.
